# Effect of Curcumin Supplementation on Physiological Fatigue and Physical Performance in Mice

**DOI:** 10.3390/nu7020905

**Published:** 2015-01-30

**Authors:** Wen-Ching Huang, Wan-Chun Chiu, Hsiao-Li Chuang, Deh-Wei Tang, Zon-Min Lee, Li Wei, Fu-An Chen, Chi-Chang Huang

**Affiliations:** 1 Graduate Institute of Athletics and Coaching Science, National Taiwan Sport University, Taoyuan 33301, Taiwan; E-Mail: magicpica521@gmail.com; 2 School of Nutrition and Health Sciences, Taipei Medical University, Taipei 11031, Taiwan; E-Mail: wanchun@tmu.edu.tw; 3 National Laboratory Animal Center, National Applied Research Laboratories, Taipei 11529, Taiwan; E-Mail: p650214@ms24.hinet.net; 4 Department of Tourism and Leisure Management, Vanung University, Chung-Li 32061, Taiwan; E-Mail: fisher1101@mail.vnu.edu.tw; 5 Department of Pharmacy, Kaohsiung Chang Gung Memorial Hospital, Kaohsiung 83301, Taiwan; E-Mail: zonmin@adm.cgmh.org.tw; 6 Department of Neurosurgery, Taipei Medical University—WanFang Hospital, Taipei 11696, Taiwan; E-Mail: nsweili@gmail.com; 7 Department of Pharmacy & Graduate Institute of Pharmaceutical Technology, Tajen University, Pingtung 90741, Taiwan; 8 Graduate Institute of Sports Science, National Taiwan Sport University, Taoyuan 33301, Taiwan

**Keywords:** anti-fatigue, lactate, ammonia, creatine kinase, blood urea nitrogen, forelimb grip strength, endurance

## Abstract

Curcumin (CCM) is a well-known phytocompound and food component found in the spice turmeric and has multifunctional bioactivities. However, few studies have examined its effects on exercise performance and physical fatigue. We aimed to evaluate the potential beneficial effects of CCM supplementation on fatigue and ergogenic function following physical challenge in mice. Male ICR mice were divided into four groups to receive vehicle or CCM (180 μg/mL) by oral gavage at 0, 12.3, 24.6, or 61.5 mL/kg/day for four weeks. Exercise performance and anti-fatigue function were evaluated after physical challenge by forelimb grip strength, exhaustive swimming time, and levels of physical fatigue-associated biomarkers serum lactate, ammonia, blood urea nitrogen (BUN), and glucose and tissue damage markers such as aspartate transaminase (AST), alanine transaminase (ALT), and creatine kinase (CK). CCM supplementation dose-dependently increased grip strength and endurance performance and significantly decreased lactate, ammonia, BUN, AST, ALT, and CK levels after physical challenge. Muscular glycogen content, an important energy source for exercise, was significantly increased. CCM supplementation had few subchronic toxic effects. CCM supplementation may have a wide spectrum of bioactivities for promoting health, improving exercise performance and preventing fatigue.

## 1. Introduction

The sensation of fatigue normally results from exertion beyond one’s normal ability. Fatigue is defined as an exercise induced inability to perform the expected or desired work output. In general, fatigue is characterized by three major aspects, according to physiological, psychological and disease theories [1]. Physiological fatigue results from excessive physical loading, inadequate rest, or mental strain/pressure and is further classified as central and peripheral fatigue [2]. Central fatigue could be caused or mediated by altered levels of histamine, 5-HT (serotonin), 5-hydroxyindoleacetic acid (5-HIAA), related neurotransmitter pathways, hormones, and exercise-induced cytokines [3,4]. Peripheral fatigue may involve the neuromuscular junction, excitation-contraction coupling process, and activation of the contractile elements involving power generation. Contractile force could decrease with interruption in any of these processes [5].

Many kinds of supplements are available for enhancing exercise performance [6], mitigating fatigue [7,8], or efficient recovery after exercise [9]. High-intensity or exhaustive physical exercises affect the body’s homeostasis, such as redox status and decrease physiological functions. Muscular injury or damage is caused by physical or chemical mechanisms. Reactive oxygen species (ROS) have been suggested to be implicated in oxidative skeletal muscle fatigue via cell membrane integrity damage by lipid peroxidation. The leakage of cells releases specific cytosol enzymes or proteins, such as creatine kinase, myoglobin, aspartate aminotransferase, and alanine aminotransferase into the blood, for muscular damage indexes [10]. Other biomarkers, such as lactate, ammonia, blood urea nitrogen (BUN), and glucose, are widely used to evaluate fatigue [11,12,13]. The diversity of nutrients or compounds from food factors or medical herbs could be investigated for their possible effect on exercise physiology and for understanding the different bioactivities that could be used for health promotion.

Curcumin (CCM) is a hydroxycinnamic acid derivative and its structure contains two hydrophobic polyphenolic rings with two carbonyl groups. It is the main curcuminoid found in the spice turmeric, a plant alkaloid obtained from the ground rhizome of the perennial herb *Curcuma longa*. It has been used in the alternative medicine systems of India (Ayurvedic medicine) and Asia (traditional Chinese medicine) to treat gastrointestinal, pulmonary and liver disorders, wounds and sprains [14,15]. In addition to medical use, it gives a distinctive flavor to curry when used as a condiment and is also used as a colorant. CCM has been studied for multiple bio-functional activities, including anti-obesity [16], anti-inflammation [17], anti-cancer [18], anti-angiogenesis [19], anti-diabetes [20], hepato-protection [21], radio-protection [22] and chemopreventive [23] activities. As well, CCM may have an effect on adiposity and lipid metabolism via several mechanisms including modulation of energy metabolism, inflammation, and suppression of angiogenesis [24,25].

However, few studies have directly addressed the effect of CCM on physiological fatigue, especially peripheral fatigue. We aimed to evaluate the potential benefits of CCM supplementation in a mouse model of physical performance test and exhaustive swimming. According to previous activities regarding lipid and energy metabolism, we hypothesized that CCM supplementation may mediate exercise-induced metabolites, energy distribution, and even physical performance. Therefore, we evaluated the potential ergogenic and anti-fatigue effects of CCM by using our previously established *in vivo* platform [7,8,11].

## 2. Experimental Section

### 2.1. Materials

CCM supplementation was prepared by good manufacturing practices and provided by Professor Fu-An Chen (Tajen University, Taiwan). The dose of CCM designed for mice was based on a daily-recommended dose of CCM at 60 mL/serving/day for humans. The mouse CCM dose (12.3 mL/kg) we used was converted from a human equivalent dose (HED) based on body surface area by the following formula from the US Food and Drug Administration: assuming a human weight of 60 kg, the HED for 60 (mL)/60 (kg) = 1 × 12.3 = a mouse dose of 12.3 mL/kg; the conversion coefficient 12.3 was used to account for differences in body surface area between mice and humans as we described previously [26].

The total curcuminoid content of CCM, including curcumin, demethoxycurcumin and bisdemethoxycurcumin, was determined by a high-performance liquid chromatography (HPLC) method described previously [27] with some modification. A Hitachi L-2130 HPLC pump system equipped with an L-2450 diode array detector and L-2200 autosampler was used to analyze curcuminoids on an Ascentis Express C18 column (i.d. 4.6 × 250 mm) at 425 nm. A mixture of acetonitrile/2% glacial acetic acid in H_2_O (50:50, v/v) was used as the mobile phase at a flow rate of 1 mL/min and an injection volume of 50 μL.

### 2.2. Animals and Treatment

Male ICR mice (6 weeks old) with specific pathogen-free conditions were purchased from BioLASCO (A Charles River Licensee Corp., Yi-Lan, Taiwan). The experimental animals were given 2 weeks to acclimatize to the environment and diet. All animals were fed a chow diet (No. 5001; PMI Nutrition International, Brentwood, MO, USA) and distilled water *ad libitum*, and maintained at a regular cycle (12-h light/dark) at room temperature (23 ± 2 °C) and 50%~60% humidity. The bedding was changed and cleaned twice per week. All animal experimental protocols were approved by the Institutional Animal Care and Use Committee (IACUC) of National Taiwan Sport University, and the study conformed to the guidelines of the protocol IACUC-10309 approved by the IACUC ethics committee.

All animals were randomly assigned to 4 groups (10 mice/group) for oral gavage treatment with CCM once a day for 28 consecutive days: vehicle (water) and CCM at 12.3 (CCM-1X), 24.6 (CCM-2X), and 61.5 mL/kg (CCM-5X). The vehicle group received the same volume of solution equivalent to body weight (BW). The food intake and water consumption were monitored daily, and BW was recorded weekly.

### 2.3. Forelimb Grip Strength

A low-force testing system (Model-RX-5, Aikoh Engineering, Nagoya, Japan) was used to measure the forelimb grip strength of mice as we previously described [7,8,11].

### 2.4. Swimming Exercise Performance Test

The swim-to-exhaustion exercise test involved mice carrying constant loads corresponding to 5% BW to analyze endurance time as we previously described [7,8,11]. The swimming endurance time was recorded from the beginning of the test to exhaustion, determined by loss of coordinated movements and failure to return to the surface within 7 s.

### 2.5. Determination of Blood Biochemical Variables Related to Physical Fatigue and Tissue Injury

The effect of CCM supplementation on levels of serum lactate, ammonia, glucose, BUN, aspartate transaminase (AST), alanine transaminase (ALT), and creatinine kinase (CK) was evaluated immediately after exercise. At 1 h after the last treatment, mice underwent a 15-min swimming test without weight loading. After the swimming exercise, blood samples were immediately collected from the submandibular duct of mice and centrifuged at 1500× *g* and 4 °C for 10 min for serum preparation. Lactate, ammonia, glucose, BUN, AST, ALT, and CK levels in the serum were determined by use of an autoanalyzer (Hitachi 7060, Hitachi, Japan) on the same day.

### 2.6. Clinical Biochemical Profile Assay

At the end of experiment, all mice were killed by 95% CO_2_ asphyxiation, and blood was immediately collected. Serum was separated by centrifugation, and the clinical biochemical variables above, plus lactate dehydrogenase (LDH), albumin, total protein (TP), creatinine, uric acid (UA), total cholesterol (TC), triglycerides (TG) and glucose levels, were measured by use of an autoanalyzer (Hitachi 7060, Hitachi, Japan).

### 2.7. Tissue Glycogen Determination

After blood collection, liver and muscle tissues were excised from mice and weighed for glycogen content analysis. The method of glycogen analysis was as we previously described [7].

### 2.8. Histological Staining of Tissues

Liver, kidney, heart, and muscle tissue was collected and immediately fixed in 10% formalin after being weighed. Heart tissue was cut transversely or longitudinally to obtain ventricular sections or four-chamber cross-sections, respectively. Tissue was then embedded in paraffin and cut into 4-μm thick slices, then stained with hematoxylin and eosin (H&E) and examined under a light microscope equipped with a CCD camera (BX-51, Olympus, Tokyo) by a clinical pathologist as we previously described [7].

### 2.9. Statistical Analysis

All data are expressed as mean ± SEM. Differences among groups were analyzed by one-way ANOVA and the Cochran–Armitage test for dose-effect trend analysis with SAS 9.0 (SAS Inst., Cary, NC, USA). *p* < 0.05 was considered statistically significant.

## 3. Results

### 3.1. Content of Curcuminoids in CCM

**Figure 1:**
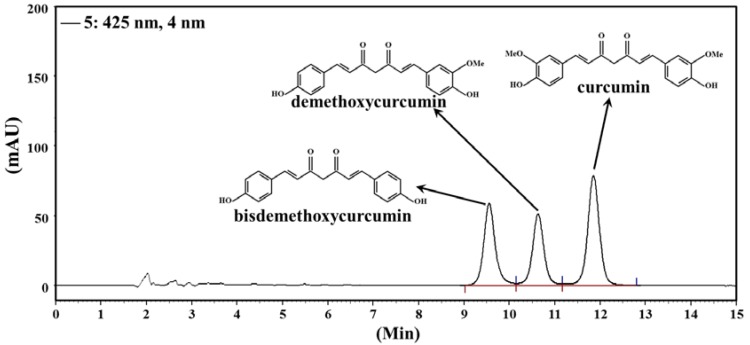
HPLC chromatogram of curcuminoids in CCM.

The retention time of curcumin, demethoxycurcumin and bisdemethoxycurcumin in CCM was 11.85, 10.63 and 9.55 min, respectively (Figure 1). The content of total curcuminoids in CCM was 180 μg/mL based on a calibration curve.

### 3.2. Effect of CCM Supplementation on Forelimb Grip Strength

**Figure 2:**
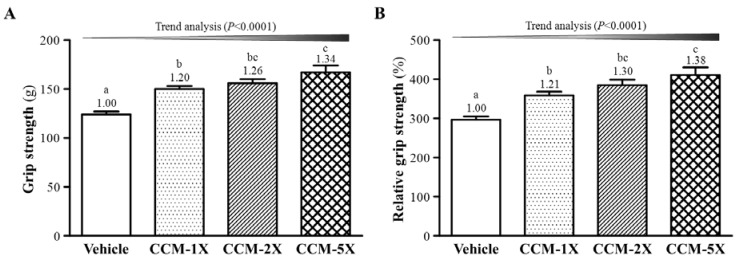
Effect of CCM supplementation on forelimb grip strength. (**A**) grip strength; (**B**) relative grip strength. Data are mean ± SEM for *n* = 10 mice in each group. Different letters indicate significant difference at *p* < 0.05 by one-way ANOVA. Low-dose (CCM-1X), medium-dose (CCM-2X) and high-dose (CCM-5X) CCM at 12.3, 24.6 and 620 mg/kg/day, respectively.

Grip strength significantly differed among treatment groups (F(3,36) = 15.53, *p* < 0.05, η^2^ = 0.564). Grip strength was significantly higher, by 1.20-, 1.25- and 1.34-fold (all *p* ≤ 0.01) with CCM-1X, CCM-2X, and CCM-5X treatment, respectively, than vehicle treatment (Figure 2A). Grip strength dose-dependently increased with CCM supplementation on trend analysis (*p* < 0.0001). Mouse weight, age, body mass index and waist circumference are associated with grip strength [28]. Therefore, grip strength was calibrated by individual BW to obtain relative grip strength (%) and was still lower with vehicle than CCM treatment (F(3,36) = 14.02, *p* < 0.05, η^2^ = 0.538, Figure 2B) with significant trend findings (*p* < 0.0001).

### 3.3. Effect of CCM Supplementation on Exhaustive Swimming Test

**Figure 3:**
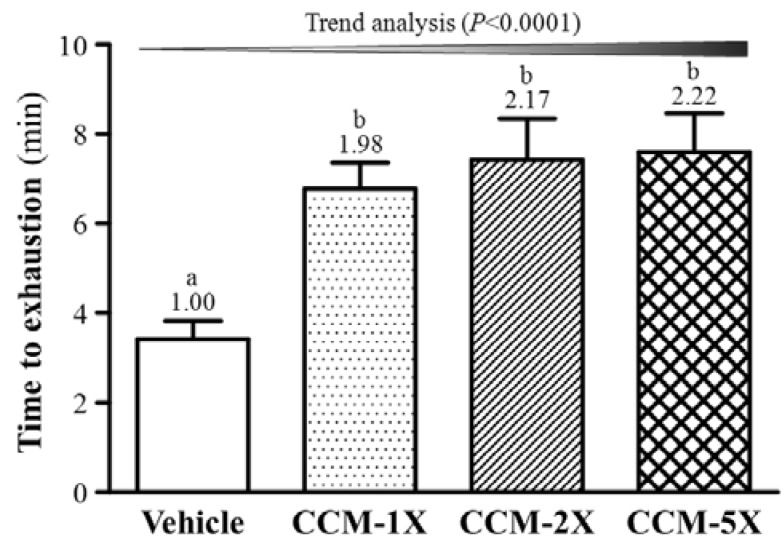
Effect of CCM supplementation on exhaustive swimming test. Data are mean ± SEM for *n* = 10 mice in each group. Different letters indicate significant difference at *p* < 0.05 by one-way ANOVA.

Exercise endurance is an important variable in assessing anti-fatigue effects. We evaluated exercise endurance ability by an exhaustive swimming test (Figure 3). Exercise endurance significantly differed among CCM treatments (F(3,36) = 7.39, *p* < 0.05, η^2^ = 0.381) The swimming time was significantly longer by 1.98-, 2.17- and 2.22-fold (all *p* < 0.01) with CCM-1X, CCM-2X, and CCM-5X treatment, respectively, than vehicle treatment.

### 3.4. Effect of CCM Supplementation on Exercise Fatigue-related Indicators after Acute Exercise

**Figure 4:**
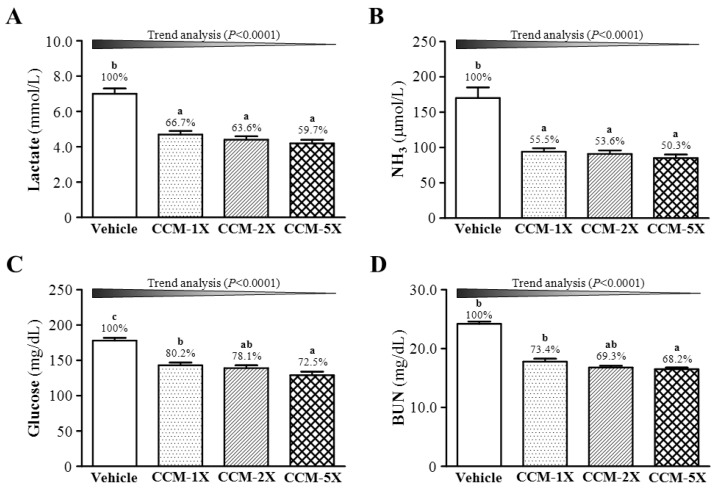
Effect of CCM supplementation on serum (**A**) lactate; (**B**) ammonia; (**C**) glucose; and (**D**) blood urea nitrogen (BUN) levels after acute exercise challenge. Data are mean ± SEM for *n* = 10 mice in each group. Different letters indicate significant difference at *p* < 0.05 by one-way ANOVA.

The status of muscle fatigue after exercise can be evaluated by important biochemical indicators, including lactate, ammonia, glucose, and BUN, after exercise [11,12,13]. We found serum lactate level lower, by 33.5%, 36.7% and 40.5% (all *p* < 0.05), with CCM-1X, CCM-2X, and CCM-5X treatment, respectively, than vehicle treatment (Figure 4A), and differed among CCM treatments (F(3,36) = 24.91, *p* < 0.05, η^2^ = 0.674). Serum ammonia level was lower, by 44.6%, 46.5%, and 49.8% (all *p* < 0.0001), respectively (Figure 4B), and differed among CCM treatments (F(3,36) = 21.79, *p* < 0.05, η^2^ = 0.644). Both lactate and ammonia levels showed a significant dose-dependent effect of CCM supplementation on trend analysis (*p* < 0.0001).

Exercise and muscle contractions increase glucose uptake by skeletal muscles by a mechanism independent of the insulin-signaling pathway [29]. During exercise, efficient utilization of glucose is an important index for performance maintenance. Serum glucose level was lower with CCM treatments than vehicle treatment after exercise and differed among CCM treatments (F(3,36) = 21.97, *p* < 0.05, η^2^ = 0.647) (Figure 4C). Glucose could be efficiently used by tissue, as seen by a significant dose-dependent effect of CCM treatment on trend analysis (*p* < 0.0001). BUN is an important metabolite caused by protein degradation after intensive exercise. BUN level was lower with CCM treatments than vehicle treatment after exercise (Figure 4D) and significantly differed among CCM treatments (F(3,36) = 98.90, *p* < 0.05, η^2^ = 0.891). BUN level after exercise decreased 26% to 31% (*p* < 0.05), for a significant dose-dependent effect on trend analysis (*p* < 0.0001).

### 3.5. Effect of CCM Supplementation on Exercise-Induced Injury Indicators after Acute Exercise Challenge

**Figure 5:**
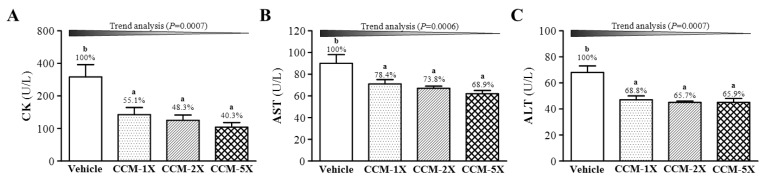
Effect of CCM supplementation on serum levels of (**A**) creatine kinase (CK); (**B**) aspartate transaminase (AST); and (**C**) alanine transaminase (ALT) after acute exercise challenge. Data are mean ± SEM for *n* = 10 mice in each group. Different letters indicate significant difference at *p* < 0.05 by one-way ANOVA.

For incremental exhaustive exercise, several indicators are used to evaluate muscle and liver injury, such as CK, LDH, AST, and ALT [12]. We assessed the effect of CCM supplementation on CK, AST, and ALT levels after acute exercise challenge. CK level significantly differed among CCM treatments (F(3,36) = 8.04, *p* < 0.05, η^2^ = 0.401) (Figure 5A) and was lower, by 45%, 52% and 60% (all *p* < 0.001), with CCM-1X, CCM-2X, and CCM-5X treatments, respectively, than vehicle treatment. AST level significantly differed among CCM treatments (F(3,36) = 6.27, *p* < 0.05, η^2^ = 0.343) and was lower, by 21% to 31% (all *p* < 0.01) with CCM treatments than vehicle treatment. ALT level significantly differed among CCM treatments (F(3,36) = 11.92, *p* < 0.05, η^2^ = 0.498) and was lower, by 30% to 33% (all *p* < 0.05) with CCM treatments than vehicle treatment (Figure 5C). CK, AST, and ALT levels showed significant dose-dependent effects on trend analysis (all *p* < 0.001).

### 3.6. Effect of CCM Supplementation on Hepatic and Muscular Glycogen Level

**Figure 6:**
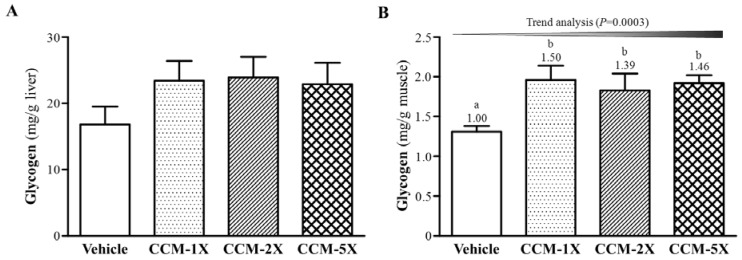
Effect of CCM supplementation on glycogen content in (**A**) liver and (**B**) muscle. Data are mean ± SEM for *n* = 10 mice in each group. Different letters indicate significant difference at *p* < 0.05 by one-way ANOVA.

Energy consumption and deficiency can lead to physical fatigue during exercise. Carbohydrates are considered the main sources of energy during exercise, and glycogen is the predominant source of glycolysis for energy production. We assessed these variables in mouse liver and muscle after exercise to understand the effect of CCM supplementation on glycogen status. Liver glycogen level did not differ among CCM treatments (F(3,36) = 1.25, *p* = 0.307) (Figure 6A). However, muscle glycogen level significantly differed among CCM treatments (F(3,36) = 3.86, *p* = 0.017, η^2^ = 0.243) (Figure 6B). Muscle glycogen level was significantly increased by 1.39- to 1.49-fold with CCM supplementation (all *p* < 0.05) compared to vehicle treatment and showed significant dose-dependent effects of treatment on trend analysis (*p* = 0.0003).

### 3.7. Subacute Toxicity of CCM Supplementation with General Characteristics

**Figure 7:**
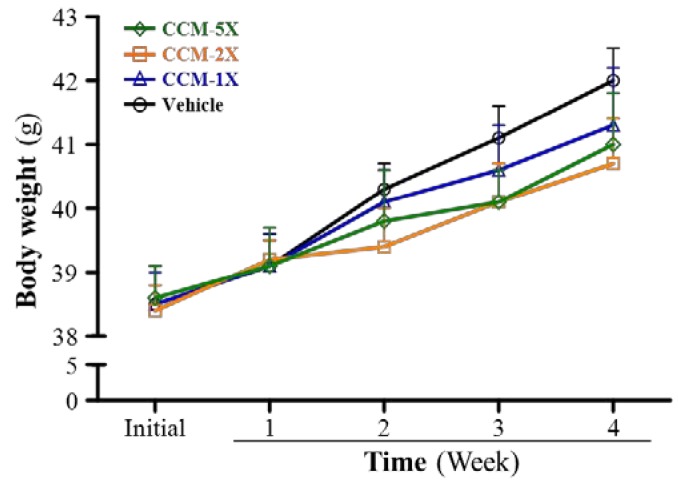
The effect of CCM supplementation on growth. Data are mean ± SEM for *n* = 10 mice per group.

We evaluated general characteristics of mice, such as behavior, growth, food intake, and organ weight, with gradual CCM supplementation. BW did not differ with all treatments and duration (four weeks) (Figure 7). The vehicle and CCM supplementation groups did not differ in behavior, such as activities and socialization, or food intake during daily observation. Epididymal fat pad (EFP) and heart tissue weights significantly differed among CCM treatments (F(3,36) = 6.841, *p* = 0.017, η^2^ = 0.363) and were lower, by 33%, 23% and 39% (all *p* < 0.05), with CCM treatments than vehicle treatment and in relative percentage (%) (Table 1). Weight of liver, kidney, lung, and muscle did not differ among treatments. Heart weight showed significant differences among treatment groups (F(3,36) = 4.018, *p* = 0.015, η^2^ = 0.250) and was lower, by 11% and 13% (both *p* < 0.05), with CCM-2X and CCM-5X treatments, respectively, than vehicle treatment. After calibration for individual body weight, CCM supplementation still significantly decreased EFP (%) weight, with significant dose-dependent effects (*p* = 0.0019). However, relative heart weight (%) was significantly decreased only with 5X treatment as compared with other treatments (*p* = 0.0484).

**Table 1:** General characteristics of the experimental groups with curcumin supplementation.

Characteristic	Vehicle	CCM-1X	CCM-2X	CCM-5X	Trend Analysis
Initial BW (g)	39 ± 0.6	39 ± 0.5	38 ± 0.4	39 ± 0.5	0.9655
Final BW (g)	42 ± 0.5	41 ± 0.9	41 ± 0.7	41 ± 0.8	0.2490
Food intake (g/day)	7.3 ± 0.1	7.4 ± 0.1	7.4 ± 0.1	7.6 ± 0.1	0.1680
Water intake (mL/day)	9.3 ± 0.2	9.4 ± 0.3	9.4 ± 0.3	9.3 ± 0.3	0.8886
Liver (g)	2.28 ± 0.05 ^b^	2.06 ± 0.06 ^a^	2.09 ± 0.05 ^a^	2.15 ± 0.05 ^a,b^	0.1192
Muscle (g)	0.39 ± 0.01	0.40 ± 0.01	0.39 ± 0.01	0.41 ± 0.01	0.3369
Kidney (g)	0.73 ± 0.03	0.73 ± 0.02	0.71 ± 0.01	0.73 ± 0.04	0.6473
Heart (g)	0.24 ± 0.01 ^b^	0.24 ± 0.01 ^b^	0.21 ± 0.01 ^a^	0.21 ± 0.01 ^a^	0.0014
Lung (g)	0.23 ± 0.01	0.22 ± 0.01	0.22 ± 0.01	0.23 ± 0.01	0.3283
EFP (g)	0.70 ± 0.05 ^b^	0.47 ± 0.04 ^a^	0.54 ± 0.04 ^a^	0.42 ± 0.04 ^a^	0.0016
Relative liver weight (%)	5.55 ± 0.14 ^b^	4.98 ± 0.08 ^a^	5.15 ± 0.18 ^a,b^	5.47 ± 0.15 ^b^	0.7800
Relative muscle weight (%)	0.93 ± 0.03	0.97 ± 0.03	0.97 ± 0.02	0.99 ± 0.02	0.0410
Relative kidney weight (%)	1.74 ± 0.07	1.80 ± 0.05	1.69 ± 0.06	1.78 ± 0.08	0.9644
Relative heart weight (%)	0.58 ± 0.03 ^b^	0.59 ± 0.03 ^b^	0.53 ± 0.02 ^a,b^	0.51 ± 0.01 ^a^	0.0060
Relative lung weight (%)	0.55 ± 0.02	0.54 ± 0.01	0.53 ± 0.02	0.55 ± 0.01	0.9823
Relative EFP weight (%)	1.65 ± 0.08 ^c^	1.13 ± 0.09 ^a,b^	1.33 ± 0.11 ^b^	1.03 ± 0.09 ^a^	0.0019

^1^ Data are the mean ± SEM for *n* = 10 mice in each group. Values in the same row with different superscript letters (^a^, ^b^) differ significantly, *p* < 0.05, by one-way ANOVA; ^2^ Muscle mass includes both gastrocnemius and soleus muscles in the back part of the lower legs. BW: body weight; EFP: epididymal fat pad. Low-dose (CCM-1X), medium-dose (CCM-2X) and high-dose (CCM-5X) CCM at 12.3, 24.6 and 620 mg/kg/day, respectively.

### 3.8. Subacute Toxicity of CCM Supplementation with Biochemistry and Histopathology Evaluation

We evaluated liver and kidney-related indices to assess physiological status after four-week CCM supplementation (Table 2). Liver-related indexes, AST and ALT, were significantly affected by CCM supplementation. AST level was lower with CCM-5X supplementation than vehicle treatment (*p* = 0.0176) and ALT level was lower, by 13%, 16% and 15% (all *p* < 0.05), with CCM treatments than vehicle treatment. The kidney-related indexes creatinine, BUN, and UA were affected by CCM supplementation. Creatinine level was lower, by 18%, 23% and 13% (all *p* = 0.01) with CCM treatments than vehicle treatment. BUN level was lower, by 10%, 16% and 17% (all *p* ≤ 0.0001), with CCM treatments and UA level was lower, by 21% and 30% (both *p* < 0.01) with CCM-2X and CCM-5X treatment, respectively, than vehicle treatment. TC level was lower, by 13% and 12% (*p* < 0.05), with CCM-2X and CCM-5X treatment, respectively, than vehicle treatment. TG level was lower only with CCM-5X treatment (*p* = 0.0234). AST, creatinine, BUN, TC, TG, and UA values exhibited significant dose-dependent effects on trend analysis (*p* < 0.05).

**Table 2:** Biochemical analysis at the end of treatment.

Parameter	Vehicle	CCM-1X	CCM-2X	CCM-5X	Trend Analysis
AST (U/L)	74 ± 4 ^b^	68 ± 4 ^a,b^	64 ± 3 ^a,b^	61 ± 3 ^a^	0.0077
ALT (U/L)	50 ± 3 ^b^	44 ± 2 ^a^	42 ± 2 ^a^	42 ± 2 ^a^	0.0507
LDH (U/L)	316 ± 23	304 ± 33	284 ± 18	278 ± 16	0.2681
Albumin (g/dL)	3.50 ± 0.05	3.51 ± 0.04	3.46 ± 0.04	3.55 ± 0.04	0.7972
TP (g/dL)	4.80 ± 0.02	4.76 ± 0.04	4.76 ± 0.04	4.83 ± 0.03	0.6559
BUN (mg/dL)	24.9 ± 0.6 ^c^	22.2 ± 0.5 ^b^	20.9 ± 0.5 ^a,b^	20.6 ± 0.4 ^a^	<0.0001
Creatinine (mg/dL)	0.23 ± 0.01 ^b^	0.19 ± 0.01 ^a^	0.18 ± 0.01 ^a^	0.20 ± 0.01 ^a^	0.0392
CK (U/L)	173 ± 15	170 ± 22	150 ± 29	151 ± 19	0.1268
UA (mg/dL)	1.58 ± 0.10 ^c^	1.36 ± 0.07 ^b,c^	1.27 ± 0.07 ^a,b^	1.12 ± 0.07 ^a^	<0.0001
TC (mg/dL)	159 ± 5 ^b^	145 ± 5 ^a,b^	139 ± 7 ^a^	140 ± 5 ^a^	0.0043
TG (mg/dL)	141 ± 11 ^b^	129 ± 9 ^a,b^	129 ± 13 ^a,b^	108 ± 6 ^a^	0.0012
Glucose (mg/dL)	179 ± 6	168 ± 3	170 ± 4	179 ± 5	0.7645

Data are mean ± SEM for *n* = 10 mice per group. Values in the same row with different superscript letters (a, b) differ significantly, *p* < 0.05, by one-way ANOVA.

**Figure 8:**
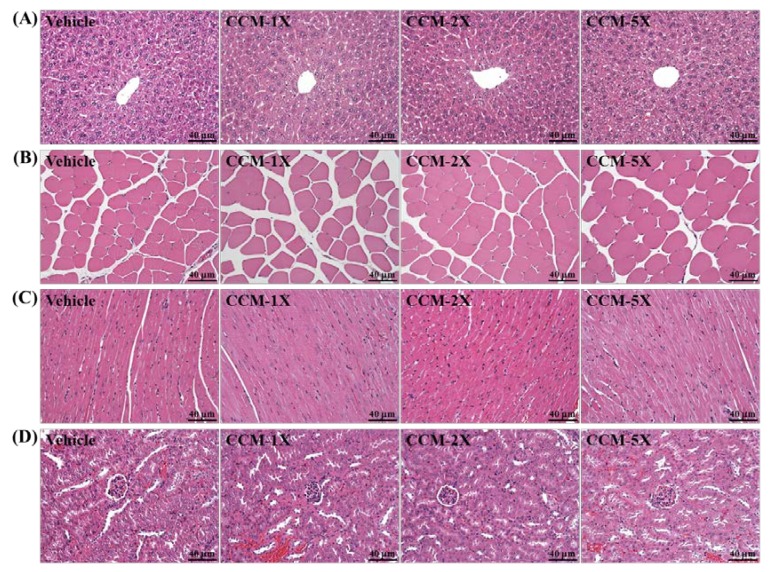
Effect of CCM supplementation on morphology of (**A**) liver; (**B**) skeletal muscle; (**C**) heart; and (**D**) kidney in mice. Specimens were photographed by light microscopy. (H&E stain, magnification: ×200; Scale bar, 10 μm).

On morphological observation, the arrangement of sinusoid and hepatic cords in liver showed no changes with CCM treatment (Figure 8). Hypertrophy and hyperplasia were not observed in heart cardiomyocytes and rhabdomyocytes of gastrocnemius muscle. In addition, the structure of renal tubules and glomerulus did not differ among treatments.

## 4. Discussion

Few studies have examined the effect of curcumin on exercise performance and physical fatigue. We aimed to evaluate the potential beneficial effects of CCM supplementation on fatigue and ergogenic function after physical challenge in mice. We evaluated exercise performance and anti-fatigue function by forelimb grip strength, exhaustive swimming time, and levels of physical fatigue-associated biomarkers. CCM supplementation dose-dependently increased grip strength and endurance and significantly decreased lactate, ammonia, BUN, AST, ALT, and CK levels after physical challenge. Muscular glycogen content, an important energy source for exercise, was significantly increased. CCM supplementation had few subchronic toxic effects. CCM supplementation may have a wide spectrum of bioactivities for promoting health and performance improvement and preventing fatigue.

In sport science, exercise endurance is usually evaluated by two different aerobic exercise models, including treadmill running and exhaustive swimming test in animals. The treadmill test forces experimental animals to run according to an increased gradient of speed or slope by negative motivation, for example electric shock, until exhaustion [12]. The other test is a swimming test by loading a mass equivalent to a percentage of body weight on the tail for exhaustive swimming based on animal survival instinct [7]. The grip strength assessment reflects the overall health of the musculoskeletal system and can also be used to evaluate motor-associated coordination and adaption in neurological study [30]. We found that grip strength could be dose-dependently increased with CCM supplementation (Figure 2). In previous reports, CCM had a neurite-promoting effect [31] and may help motor units efficiently recruit fiber for higher power output. Therefore, the regulatory training program combined with CCM supplementation may accelerate musculoskeletal adaption and coordination in future practice and application. For endurance capacity assessed by exhaustive swimming test, exhaustive endurance was dose-dependently extended with CCM supplementation (Figure 3). Reports of bio-functional activities of the effect of curcumin on exercise performance are limited, and most focused on anti-oxidative activity. The capacity of aerobic exercise is highly associated with cardiovascular function. In previous reports, curcumin could possibly improve cardiovascular health by ameliorating postprandial endothelial function (flow-mediated vasodilation) in healthy subjects [32] and anti-oxidative stress induced by treadmill running [33]. Central fatigue also plays an important role in performance during prolonged exercise, and curcumin was reported to modulate central neuronal monoaminergic neurotransmission, especially transmission of serotonin [34]. In addition, curcumin could improve exercise performance by reducing inflammation and exercise-induced muscle damage [35]. Therefore, endurance could be increased because of improved cardiovascular function and anti-oxidation activity and modulation of monoaminergic neurotransmission, exercise-induced inflammation and damage.

We have limited reports of the effect of curcumin on exercise-induced fatigue. Here, we focused on important serum indicators, such as lactate, ammonia, glucose, CK, BUN, ALT, and ALT, related to exercise fatigue or injury [11,12,13]. During intensive/prolonged exercise, muscles must obtain immediate/sufficient energy from anaerobic glycolysis, and abundant lactate is produced and accumulated by glycolysis metabolism. The increased lactate level results in decreased pH value, which could have deleterious effects on glycolysis, enzyme activities, and muscular contractions due to calcium ion release [36]. The level of another important metabolite, ammonia, significantly increases with intensity or prolonged time during exercise. It is substantially elevated during intensive or prolonged exercise when the rate of ATP utilization may exceed that of ATP production. Ammonia toxicity may affect continuing coordinated activity in critical regions of the central nervous system [37]. Acute exercise can increase the production of oxidative stress factors, reactive oxygen species, and induce muscle or liver damage especially with exhaustive exercise [12,38]. For the first time, we assessed the effect of CCM on related indicators and found that it could significantly decrease the levels of physical fatigue-associated metabolites or exercise-induced injury indicators (Figure 4 and Figure 5).

Energy expenditure is also an important issue to maintain exercise capacity. Glucose homeostasis is regulated by tissue glycogen and is a limiting factor in prolonged exercise. However, nutritional interventions could be beneficial to modulate tissue glycogen content before or during exercise [39]. We found that CCM supplementation could significantly increase glycogen content in muscle rather than liver tissue (Figure 6). Curcumin treatment protected against regional myocardial ischemia/reperfusion injury in the heart by activating prosurvival kinases such as glycogen synthase kinase-3 [40]. Therefore, CCM supplementation may have different roles for glycogen modulation in different tissues for related functional activities. We found that CCM could significantly extend endurance in mice by regulating exercise-fatigue or injury-related indicators and energy supply from muscle glycogen.

Safety is a concern when considering the use of specific extracts or herbs as plant-derived nutritional, medicinal or health-care products. We lack comprehensive subacute safety studies of CCM. We assessed its subacute toxic effects by mouse behavior, weight (growth), diet, organ weight, clinical biochemistry and histopathology with 28-day CCM repeated-dose supplementation. Previously, CCM toxicity did not result in any significant treatment-related changes in behavior, growth, weight, food consumption, organ weight, hematology, clinical biochemistry, and urinalysis [41]. However, we found that CCM had beneficial effects on organ weight and clinical biochemistry variables. CCM supplementation significantly decreased the weight of fat tissue, EFP, and levels of lipid-related parameters, TG and TC. Previous reports showed that curcumin treatment reduced liver TG levels [42] and exhibited hypocholesterolemic effects by increasing the expression of ApoAI, CYP7A1, lecithin:cholesterol acyltransferase and low-density lipoprotein receptor [43]. In a previous study of its protective effect on the kidney, curcumin prevented the decrease in nuclear factor (erythroid-derived 2)-like and heme oxygenase 1 expression in the remnant kidney and ameliorated renal dysfunction in a nephrectomized rat model [44]. We also found that CCM could improve parameters related to kidney functions (Table 2). Histological data related to the pathological effects of CCM is quite limited, especially the indicated bioactivity doses. We found no changes in arrangement of sinusoid and hepatic cords with CCM treatment in mice and no hypertrophy or hyperplasia response in heart cardiomyocytes and rhabdomyocytes of gastrocnemius muscles. We found no significant difference in structure of renal tubules and glomerulus with CCM treatment and no significant alteration in the alveolar, bronchial and interstitial space.

## 5. Conclusions

Here we found that CCM supplementation had significant benefits for physiological indicators after exercise and improved exercise performance, including grip strength and endurance, by increasing muscle glycogen content. In addition, it also showed benefit effects on body composition and biochemistry on lipid profiles, liver, and rental parameters. The toxicity of CCM was elucidated as safety in relevant observations. Therefore, CMM could help ameliorate exercise-induced fatigue and contribute to health promotion safely.
